# The Instrumental Role of Strategic Communication to Counter Industry Marketing Responses to Sugary Drink Taxes: Comment on "Understanding Marketing Responses to a Tax on Sugary Drinks: A Qualitative Interview Study in the United Kingdom, 2019"

**DOI:** 10.34172/ijhpm.2023.7685

**Published:** 2023-02-28

**Authors:** Nandita Murukutla, Trish Cotter, Alexey Kotov

**Affiliations:** Policy Advocacy and Communication Division, Vital Strategies, New York City, NY, USA.

**Keywords:** Sugary Drink, Sugary Drink Tax, Strategic Communication, Mass Media Campaigns, Commercial Determinants, Corporate Influence

## Abstract

Strong sugary drink taxes are effective at reducing sugary drinks consumption. In response, the sugary drinks industry employs various marketing strategies to undermine the taxes to protect and maintain its customer base. In their recent article in this journal, Forde et al present a framework for understanding how sugary drinks companies use marketing for this purpose. In this commentary, we reflect on this framework by drawing from recent experiences of sugary drinks industry marketing responses. Further, we review the global evidence on the instrumental role that strategic communication can play in protecting strong taxes from industry responses. We make a case for strategic communication as a vital tool in promoting and protecting sugary drinks tax proposals, both prior to and after their introduction.

## Introduction

 In their recent paper, Forde et al present a framework for understanding soft drinks (hereafter referred to as “sugary drinks”) companies’ marketing responses to the Sugary Drinks Industry Levy in the United Kingdom.^1^ They interviewed key stakeholders from industry, academia and civil society, to identify the industry’s post-tax responses across the “four Ps” of marketing—price, product, placement, and promotion—and how companies use this to maintain their brand identity and profits.

 We expand upon Forde and colleagues’ findings in this commentary by reviewing industry marketing responses, both before and after the introduction and implementation of taxes. We then discuss why countering industry marketing efforts through strategic communication is crucial to the success of sugary drinks taxes. Our discussion is relevant to policy-makers and public health advocates promoting taxes on health-harming products, including sugary drinks.

## The Global Fight for Sugary Drinks Taxes

 Sugary drinks are a major contributor to overweight and obesity, type 2 diabetes and cardiovascular disease, which are well-established risk factors for non-communicable diseases and leading causes of death and disability worldwide. Increasingly, sugary drinks taxes are being proven to be effective at reducing sugar consumption by raising prices to reduce consumer demand and forcing manufactures to reduce sugar content. In addition, they can generate revenue to support programs promoting healthy nutrition. Amid growing evidence of their success, more than 45 countries and local jurisdictions have now passed these taxes.^2^

 Marketing is a principal tool that commercial entities use to undercut public health policy. Companies use sophisticated marketing strategies, increasingly online, to protect their brand equity and maintain their consumer base. In addition, companies often engage in “corporate political activities” that include public relations and media campaigns, community mobilization, and direct lobbying to derail tax policies. Therefore, studying sugary drinks company marketing practices is important to public health counter-efforts.

## Marketing Responses to Sugary Drinks Taxes

 Evidence on the sugary drinks industry’s marketing response to sugary drinks taxes is still emerging. Most studies have focused on how the industry has changed the price of sugary drinks in response to taxes.^2,3^

 The bulk of this evidence shows companies increase prices of sugary drinks and pass on the tax burden to consumers. This was observed in countries with single-tier volume-based excise taxes including Denmark, France, and Mexico.^2^ In Mexico, studies have shown significant decreases in sugary drink purchases. In this respect, sugary drinks taxes have worked as intended.

 Another marketing response observed in response to sugary drinks taxes is product reformulation. This has occurred in countries, such as the United Kingdom and Portugal, that levy different taxes per tier of sugary content. In these cases, product reformulation has been both the expected and observed outcome of the tax.^2^ However, it is uncertain from a public health perspective whether reformulation should be considered a desired outcome, or a harmful industry marketing response. The longer-term effects of non-sugar sweeteners and low- or no-calorie alternatives to free sugars are unclear. Moreover, there is concern that the switch to nonnutritive sweetened sugary drinks, instead of water, maintains sweet preferences and habits. In addition, companies may use reformulation as a corporate social responsibility tactic by making it appear that they have the consumers best interest in mind and use it to support the case for voluntary actions.

 Sugary drinks companies have changed promotion tactics in response to taxes to improve sales and maintain profits. In Barbados, for example, advertising, particularly for juices, increased around the introduction of the tax.^4^ These ads emphasized health benefits and “naturalness” despite the excessive sugar content in the juices. In Mexico, sugary drinks companies used aggressive in-store promotions and marketing in response to the tax.^5^

 Marketing promotion to destabilize sugary drinks taxes has been observed both before and after tax implementation. Promotional activities include media and public relations campaigns; corporate political activities to influence policymakers; and corporate social responsibility programs to refurbish the image of the sugary drinks companies. These activities seek to undercut taxes by politicizing them; creating fear about their economic burden; and sowing doubt about their potential to improve public health outcomes.

 A messaging tactic frequently used by the industry to challenge the implementation of sugary drinks taxes is to deflect the blame away from sugary drinks to physical inactivity as the cause of obesity. This line of argumentation is used despite the preponderance of public health evidence that has identified the obesogenic environment as the primary driver of obesity. In the United States, for example, Coca-Cola funded scientists at universities to start the Global Energy Balance Network, which propagated that exercise can offset unhealthy diets.^6^ Coca-Cola also donated millions to establish fitness programs at schools in the United States and globally, and donated millions to establish fitness programs in the city of Chicago after a sugary drinks tax was proposed. Similar efforts to shift focus to physical activity were also undertaken by industry stakeholders in South Africa. These efforts do appear to work: In surveys we have conducted in both South Africa and Jamaica—where taxes were being proposed—many participants thought that physical activity can offset the harms of sugar.^7,8^

 There is likewise evidence of industry marketing promotion following tax implementation. The intent appears to be to roll back or weaken taxes and to prevent their global spread. An example of such post-tax marketing interference comes from Mexico, where sugary drinks companies mounted a concerted campaign to discredit a tax introduced in 2014. Industry-sponsored research wrongly suggested that the tax had no health benefits and harmed the economy. These findings were shared with politicians, the media, and other stakeholders to discredit the tax’s efficacy.

 While experience with industry marketing responses to sugary drinks taxes continues to build, it may be instructive for public health practitioners to consider the experience in tobacco control. Tobacco taxes have a longer history of implementation, and marketing strategies used by tobacco companies may become relevant to the sugary drinks industry as it faces increasing regulation. The study of tobacco industry responses to tax increases has noted several common strategies to avoid increasing prices; these include shifting the tax burden between products, launching new brands or products to avoid taxation, and product promotions, among others.^9^ A particular marketing tactic observed in the tobacco industry and also commented upon by Forde et al is the use of surrogate marketing — a tactic commonly observed with tobacco products and increasingly with alcohol. Interviewees in the Forde et al study mentioned that some brands repackaged lower-sugar tax-exempt products to resemble higher-sugar products. The use of surrogate marketing, where non-regulated products are used as a stand-in to promote regulated products, is a tactic that is frequently used in India by tobacco companies.

 In sum, the evidence shows that sugary drinks companies engage in a variety of marketing tactics in response to sugary drinks taxes to dampen their impact and protect profits. Fortunately, public health advocates have evidence-backed communication tools at their disposal to counter some of these efforts.

## The Important Role of Strategic Communication to Promote Sugary Drinks Taxes

 There is increasingly clear evidence that public health advocates must build—and maintain—public and stakeholder support to ensure the passage and successful implementation of sugary drinks taxes. In fact, it is particularly important to maintain support for taxes following their implementation.

 The important role of communication campaigns in helping to do this is evident from the experiences of jurisdictions in the United States that sought to pass sugary drinks taxes. In Cook County, Illinois, a tax on sugary drinks was rescinded shortly after implementation.^10^ A retrospective analysis identified anti-tax media backlash, the lack of a pro-tax campaign, and inconsistent messaging as among the reasons for this repeal. In contrast, in other US jurisdictions — specifically, Berkeley and San Francisco, California, Seattle, Washington, and Boulder, Colorado — taxes were successfully maintained.^11^ In these locations, concerted, multi-stakeholder advocacy and effective communication accompanied the introduction of taxes. These experiences underscore the importance of communication that begins prior to tax introduction and is maintained through implementation to reinforce its significance.

 Strategic communication efforts may themselves exert an independent and complementary effect on sugary drinks consumption by increasing public support for taxes and shifting social norms toward sugar consumption. In Berkeley, California, the sale of sodas on university campuses decreased immediately after the tax was adopted and even before it was implemented.^9^ Similarly, in the United Kingdom, shifts in consumption were noted even prior to the implementation of the tax there,^12^ suggesting the influence of the public debates.

 Based on these experiences, public health advocates might consider the following actions to support the successful implementation of sugary drinks taxes.

###  Implement Strategic Communication Campaigns

 Communication campaigns and other public education initiatives are an important part of a comprehensive policy package. Media campaigns can increase population-level knowledge about the harms of sugary drinks, change behaviors related to consumption and shift social norms so that sugary drinks are viewed less favorably and healthier options more favorably. Campaigns are also an important tool in the health policy development process, particularly for policies like health taxes that can be the target of considerable industry and political pushback. Campaigns have increased public and policymaker support for taxes on sugary drinks in several countries, including Jamaica and South Africa.^8,9^ They are also important to counter industry-sponsored campaigns with accurate health information.

###  Establish Support From a Diversity of Stakeholders

 Campaigns to pass sugary drinks taxes have been successful when there has been support from diverse sectors that are seen to represent a large movement. In fact, advocacy coalitions that have successfully campaigned for sugary drinks taxes in Mexico and South Africa have engaged a diversity of stakeholders, including academics, civil society organizations, and the media.

 Commercial stakeholders may also serve as allies. Despite industry claims that retailers incur profit and employment losses, a study of retailers in three cities in California found that they were largely supportive of sugary drinks taxes and reported minimal to no effects of the tax on their overall business.^13^ In Mexico, vendors found drops in soda sales, but increases in the sale of bottled water and other less sugary beverages.^5^

 The media is a particularly important stakeholder. While media backlash can lead sugary drinks tax implementation to fail, favorable media coverage can build support and counter industry opposition arguments. In the United Kingdom for example, public health advocates were prominently featured in newspapers making pro-tax arguments that focused on health effects of sugary drinks and the culpability of the industry, during the period leading up to the introduction of the tax. These efforts led to more pro-tax than anti-tax coverage, which helped shape opinions.^14^

###  Frame the Issue So Key Stakeholders Will Care

 Messages must be framed so people will care, which is the tactic that the industry takes. For example, the industry might describe sugary drink taxes as “grocery taxes” to sow fear that more food and drink products were next, or as a “sin tax” to discredit it. Associating the industry with this anti-tax rhetoric may be effective in neutralizing its effects.

 Highlighting the “common good” of sugary drinks taxes—that they serve as a public good to reduce health burdens—may serve as an effective counterfoil to the industry framing of it as a fungible economic good. In particular, describing how the revenue generated will meet public priorities, such as strengthening public programs or addressing inequities, is effective in building public support.

## Conclusion

 The sugary drinks industry uses a range of marketing tactics to prevent and undermine taxes on sugary drinks. Their goal is to maintain a socially responsible image in the eyes of stakeholders, retain profits and impede the proliferation of taxes. To counter these activities and retain control of the narrative to help ensure the passage and successful implementation of sugary drinks taxes, public health advocates must build and maintain the support of the public and other key stakeholders. This includes running strategic communication campaigns that support the tax, as well as establishing coalitions of advocates that engage with policymakers, the media, and store retailers, which are stakeholders the industry often tries to sway. Strong and consistent messaging that focuses on the health benefits from taxes and presents them as a “public good,” is key to building this support.

## Ethical issues

 Not applicable.

## Competing interests

 Authors declare that they have no competing interests.

## Authors’ contributions

 Conception and design: NM. Drafting of the manuscript: NM, TC, and AK. Critical revision of the manuscript: NM, TC, and AK.

## Funding

 Vital Strategies’ Food Policy program is funded by Bloomberg Philanthropies. However, Bloomberg Philanthropies was not involved in the writing of this manuscript. The authors have not been paid to write this article.
